# Characterization of the Molecular Mechanisms Underlying Lurasidone‐Induced Acute Manic Episodes in Bipolar Depression: A Network Pharmacology and Molecular Docking Approach

**DOI:** 10.1111/cns.70383

**Published:** 2025-04-09

**Authors:** Chao Li, Lei Yang, Qiuyu Zhang, Ying Zhang, Ranli Li, Feng Jia, Lina Wang, Xiaoyan Ma, Hongjun Tian, Chuanjun Zhuo

**Affiliations:** ^1^ Computational Biology and Animal Imaging Center (CBAC) Tianjin Anding Hospital, Nankai University Affiliated Tianjin Anding Hospital, Tianjin Medical University Affiliated Tianjin Anding Hospital, Tianjin Medical University Affiliated Tianjin Mental Health Center Tianjin China; ^2^ Laboratory of Psychiatric‐Neuroimaging‐Genetic and Co‐Morbidity (PNGC_Lab), Tianjin Anding Hospital Tianjin Mental Health Center of Tianjin Medical University Tianjin China; ^3^ Animal Imaging Center (AIC) of Tianjin Fourth Center Hospital Tianjin Medical University Affiliated Tianjin Fourth Center Hospital, Nankai University Affiliated Tianjin Fourth Center Hospital Tianjin China

**Keywords:** acute mania, bipolar depression, lurasidone, molecular docking, network pharmacology

## Abstract

**Background:**

Lurasidone monotherapy has been approved for the treatment of bipolar depression. However, several case reports have indicated treatment with lurasidone‐induced acute mania in people with bipolar depression. The mechanism by which this occurs remains to be elucidated.

**Objective:**

In this study, we systematically explored the mechanism of action of lurasidone‐induced acute mania in bipolar depression using network pharmacology and molecular docking.

**Methods:**

Putative target genes for lurasidone were obtained from the GeneCards, PharmMapper, SwissTargetPrediction, and DrugBank databases. Targets for bipolar depression and acute mania were collected from the DisGeNET and GeneCards databases. A protein–protein interaction (PPI) network was built to screen the hub targets. The Bioinformatics platform and Database for Annotation, Visualization, and Integrated Discovery were used for the visualization of the Gene Ontology and Kyoto Encyclopedia of Genes and Genomes analyses of the top 20 core targets. The drug‐pathway‐target‐disease network was constructed using Cytoscape. Finally, molecular docking was performed to evaluate the binding affinity between lurasidone and potential targets.

**Results:**

In total, 327, 1253, and 429 targets of lurasidone, bipolar depression, and acute mania were identified, respectively. A topological analysis of the PPI network revealed the top 20 hub targets. Based on PPI, Gene Ontology, and Kyoto Encyclopedia of Genes and Genomes pathway analyses of the top 20 hub targets, lurasidone was found to induce acute manic episodes in people with bipolar depression by targeting the serotonergic synapse signaling pathway via MAOB, HTR1A, HTR2A, HTR3A, SLC18A2, HTR1B, and HTR7. Molecular docking revealed good binding affinities between lurasidone and these potential targets.

**Conclusions:**

This study revealed that lurasidone may regulate the serotonergic synapse signaling pathway by interacting with the identified core targets MAOB, HTR1A, HTR2A, HTR3A, SLC18A2, HTR1B, and HTR7 to induce treatment‐emergent mania in people with bipolar depression. Our work provides a theoretical basis for the pharmacology of lurasidone‐induced acute mania in bipolar depression and further basic research.

## Graphical abstract

This study is the first to systematically explore the molecular mechanisms underlying lurasidone‐induced acute manic episodes in people with bipolar depression using network pharmacology and molecular docking approaches. Our bioinformatics and computational analyses demonstrated that lurasidone may regulate the serotonergic synapse signaling pathway by targeting proteins such as MAOB, HTR1A, HTR2A, HTR3A, SLC18A2, HTR1B, and HTR7 to induce treatment‐emergent mania in people with bipolar depression. Our work provides a theoretical basis for the pharmacology of lurasidone‐induced acute mania in bipolar depression and further basic research.

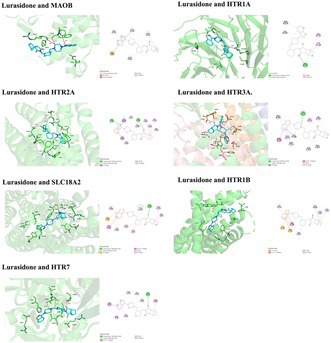

## 1 Introduction

Bipolar disorder is a chronic disorder characterized by recurrent episodes of mania/hypomania and depression, and it affects more than 1% of the global population [1, 2]. It is categorized into bipolar I and bipolar II, which DSM‐IV criteria define based on the duration and severity of elevated mood episodes. Bipolar disorder is heterogeneous and complex, including symptoms such as mixed mood states, rapid cycling, delusions and hallucinations, cognitive impairment, and reduced quality of life [3, 4, 5, 6, 7]. People with bipolar disorder commonly spend three times longer in the depressive phase than the manic phase of the illness [8]. The high prevalence, morbidity, and mortality of bipolar disorder, as well as the high burden of the illness, make its treatment an important target in psychiatry [9, 10, 11]. Although the manic episodes of bipolar disorder can commonly be effectively treated with medication (e.g., antipsychotics), recurrent depressive episodes continue to be a major challenge in clinical practice [12]. Treatment for bipolar depression with antidepressants may increase the risk of drug‐induced manic‐switch episodes. It has been widely hypothesized that disturbances in neurotransmitter systems, including serotonin [13], gamma‐aminobutyric acid [14], and glutamate [15], as well as other molecular components such as brain‐derived neurotrophic factor [16], contribute to this condition.

Lurasidone is a benzothiazol derivative approved for use in the treatment of schizophrenia and bipolar depression by the United States Food and Drug Administration [17, 18]. Lurasidone is a high‐affinity antagonist of dopamine‐2 (D_2_), alpha‐1 noradrenergic, 5‐hydroxytryptamine 2A (5‐HT_2A_), and 5‐HT_7_ receptors, as well as a partial 5‐HT_1A_ receptor agonist with negligible affinities for histamine H_1_, muscarinic M_1_, and other receptors [19]. The D2 receptor blockade of lurasidone is believed to underlie antipsychotic efficacy, and its activity on 5‐HT_1A_, 5‐HT_2A_, and 5‐HT_7_ receptors is hypothesized to improve cognition. While behavioral pharmacology studies have shown that the early anxiolytic and antidepressant effects of lurasidone are associated with the modulation of 5‐HT_1A_, alpha‐1 noradrenergic, and 5‐HT_7_ receptors [19, 20, 21, 22], the inhibition of norepinephrine reuptake is essential for its maintenance. Lurasidone's low affinity to histaminergic H1 and muscarinic M1 receptors indicates a less severe side effect profile compared to other antipsychotics, which often includes lipid abnormalities, hyperglycemia, orthostatic hypotension, anticholinergic effects, weight gain, and sedation [19]. Recently, several studies have reported that lurasidone induces acute manic episodes in people with bipolar depression [23, 24, 25]. It is speculated that this effect may be related to the excessive release of serotonin, norepinephrine, and dopamine [23, 24] However, the exact pharmacological mechanism by which lurasidone induces acute manic episodes in people with bipolar depression remains to be elucidated.

Network pharmacology, a potential approach based on bioinformatics and systems biology, is gaining increasing attention [26, 27, 28]. This approach takes full advantage of database information based on the “drug‐pathway‐target‐disease” network to clarify the effects of drugs on disease from a holistic perspective and gain a comprehensive understanding of the drug mechanism. As such, examining drug action from a network pharmacology perspective improves drug discovery for complex diseases. Molecular docking, a commonly used tool, is often used to explore the affinity between small molecules and macromolecules. Network pharmacology and molecular docking approaches have been successfully used in Western medicine to investigate the molecular mechanisms underlying and treat specific diseases [29, 30].

**FIGURE 1:**
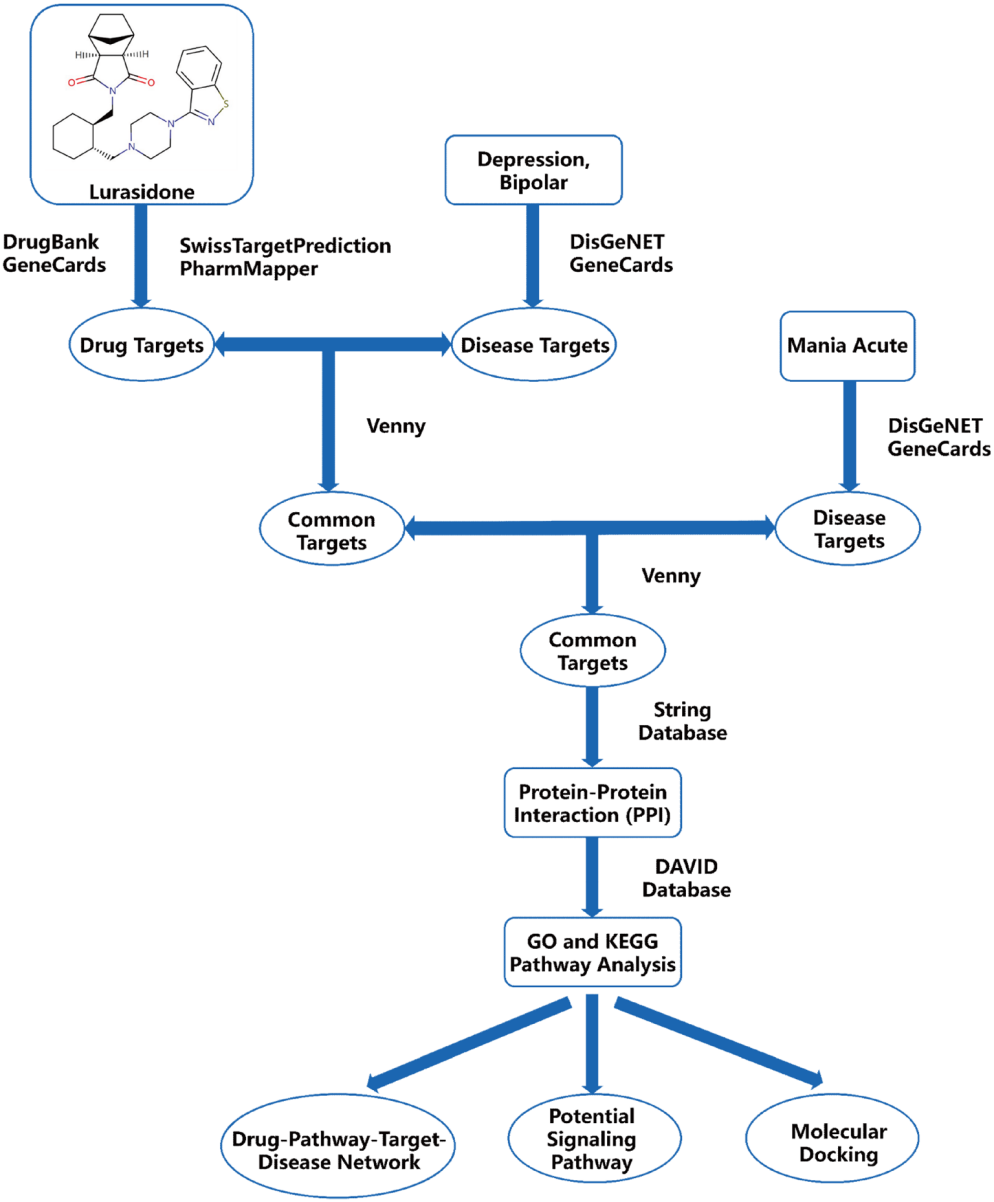
Workflow of the analyses performed in this study.

In the present study, several database resources were integrated to conduct a multilevel analysis of lurasidone‐induced acute mania in people with bipolar depression and its mechanisms of action (Figure 1). These analyses, including target identification, protein–protein interaction (PPI) network and drug‐pathway‐target‐disease network construction, and signaling pathway enrichment, contributed to a more complete understanding of the effects of lurasidone and provided a theoretical basis for its clinical application.

## 2 Methods

### 2.1 Identification of Potential Targets of Lurasidone

The chemical structure of lurasidone was obtained from the DrugBank database (https://go.drugbank.com) [31]. DrugBank, a bioinformatics and cheminformatics resource, combines detailed drug data with comprehensive information on drug targets. Potential targets of lurasidone were predicted using the GeneCards (https://www.genecards.org/) [32], PharmMapper (https://www.lilab‐ecust.cn/pharmmapper/) [33], DrugBank, and SwissTargetPrediction (http://www.swisstargetprediction.ch/) databases [34]. GeneCards is a comprehensive and user‐friendly database that allows for the search and integration of information on all annotated and predicted human genes. PharmMapper is a freely accessed web server for identifying potential targets for given small molecule probes using pharmacophore mapping. SwissTargetPrediction, founded on a combination of 2D and 3D similarity, allows for the estimation of the most likely bioactive macromolecular targets of small molecules.

### 2.2 The Collection of Bipolar Depression and Acute Mania Relevant Targets

The online databases GeneCards and DisGeNET [35] (https://www.disgenet.org/) were searched to collect disease‐related targets using the keywords “depression bipolar” and “mania acute,” respectively. DisGeNET is a discovery platform containing genes and variants associated with human diseases that integrate data from scientific literature, animal models, expert‐curated repositories, and genome‐wide association study catalogs. The GeneCards database contained a large number of targets for bipolar depression; therefore, to refine the results, the median screening method was used three times to remove targets with relevance scores below 3.7498. After combining and removing duplicates from the two databases, targets for bipolar depression and acute mania were collected.

### 2.3 Drug–Disease Target Interaction

Lurasidone‐related gene interaction targets, bipolar depression‐related targets, and acute mania‐related targets were obtained using Venny 2.1.0 (https://bioinfogp.cnb.csic.es/tools/venny). The Bioinformatics platform (http://www.bioinformatics.com.cn/) was used to construct a Venn diagram for visualization.

### 2.4 Protein–Protein Interaction (PPI) Network Construction and Hub Target Selection

The interaction targets identified from the three datasets were imported into the STRING database (https://string‐db.org) to obtain the PPI network [36]. In the operation interface, the species was restricted to “ *Homo sapiens* ,” the confidence score was set at 0.4, and the disconnected nodes were hidden. Cytoscape 3.10.0 software was used to visualize the PPI network and perform the systematic network analysis [37]. Based on the maximal clique centrality method, the CytoHubba plug‐in in Cytoscape was used to identify the top 20 hub targets [38].

### 2.5 GO Enrichment and KEGG Pathway Analyses of Hub Targets

A functional enrichment analysis tool, the Database for Annotation, Visualization and Integrated Discovery (version 2021, https://david.ncifcrf.gov/) [39], was used to perform the Gene Ontology (GO) enrichment and Kyoto Encyclopedia of Genes and Genomes (KEGG) pathway analyses of the top 20 hub targets. The GO enrichment categories included biological processes (BPs), cellular components (CCs), and molecular functions (MFs). The top 30 GO enrichment categories (10 BPs with *p* < 0.05, 10 CCs with *p* < 0.05, and 10 MFs with *p* < 0.05) and the top 10 relevant pathways (*p* < 0.05) were plotted as bar graphs and bubble plots, respectively.

### 2.6 Drug‐Pathway‐Target‐Disease Network Construction

Cytoscape 3.10.0 was used to construct the drug‐pathway‐target‐disease network to better understand the molecular mechanism underlying lurasidone‐induced acute manic episodes in people with bipolar depression. The network targets were derived from the drug‐disease interaction targets enriched in the top 10 pathways.

### 2.7 Molecular Docking Verification

Lurasidone docking was simulated with seven targets identified from the hub targets. AutoDockTools 1.5.6 and AutoDock Vina were used for molecular docking [40, 41]. The molecular docking process consists of the following steps:

#### 2.7.1 Ligand Preparation

The 3D structure file (sdf format) of lurasidone was downloaded from the PubChem database (https://pubchem.ncbi.nlm.nih.gov/) [42], opened using ChemBio3D Ultra 14.0, and saved as a pdb file. The pdb file was then processed using AutoDockTools 1.5.6 software and saved as a pdbqt file.

#### 2.7.2 Protein Preparation

The protein crystal structures were obtained from the RSCB Protein Data Bank (http://www.rcsb.org) [43] and imported into PyMOL 2.4.1 to remove water and initial ligands. Receptor proteins were hydrogenated, and their charges and atom types were calculated using AutoDockTools 1.5.6. The resulting structure was saved in the pdbqt format.

#### 2.7.3 Grid Preparation

The potential binding sites were identified using DoGSiteScorer (https://proteins.plus/), and the docking box size and coordinates were adjusted accordingly.

#### 2.7.4 Molecular Docking

Molecular docking and binding energy evaluations were performed using AutoDock Vina. The interactions between lurasidone and the core target proteins were visualized using PyMOL 2.4.1 and Discovery Studio 2021.

Table 1 provides an overview of the tools and databases used in our research, offering a clearer understanding of the specific methods and resources employed.

**TABLE 1:** Project overview and resources.

Project	Databases and tools	Sources
Target collection	GeneCards	https://www.genecards.org/
	PharmMapper	http://www.lilab‐ecust.cn/pharmmapper/
	DrugBank	https://go.drugbank.com
	SwissTargetPrediction	http://www.swisstargetprediction.ch/
	DisGeNET	https://www.disgenet.org/
Drug–disease target intersection	Venny 2.1.0	https://bioinfogp.cnb.csic.es/tools/venny
Protein–protein interaction	STRING	https://string‐db.org
	Cytoscape 3.10.0	https://www.cytoscape.org/
	CytoHubba plug‐in	https://www.cytoscape.org/
GO and KEGG enrichment analysis	DAVID	https://david.ncifcrf.gov/
	Bioinformatics platform	http://www.bioinformatics.com.cn/
Drug–pathway–target–disease network	Cytoscape 3.10.0	https://www.cytoscape.org/
Molecular docking	PubChem	https://pubchem.ncbi.nlm.nih.gov/
	AutoDockTools 1.5.6	https://autodock.scripps.edu/
	AutoDock Vina	https://vina.scripps.edu/
	PDB	http://www.rcsb.org
	PyMOL 2.4.1	https://pymol.org/
	Discovery Studio 2021	https://www.3ds.com/products/biovia/discovery‐studio

Abbreviations: DAVID, Database for Annotation Visualization and Integrated Discovery; GO, Gene Ontology; KEGG, Kyoto Encyclopedia of Genes and Genomes; PDB, Protein Data Bank.

## 3 Results

### 3.1 Targets of Lurasidone‐Induced Acute Mania in Bipolar Depression

**FIGURE 2:**
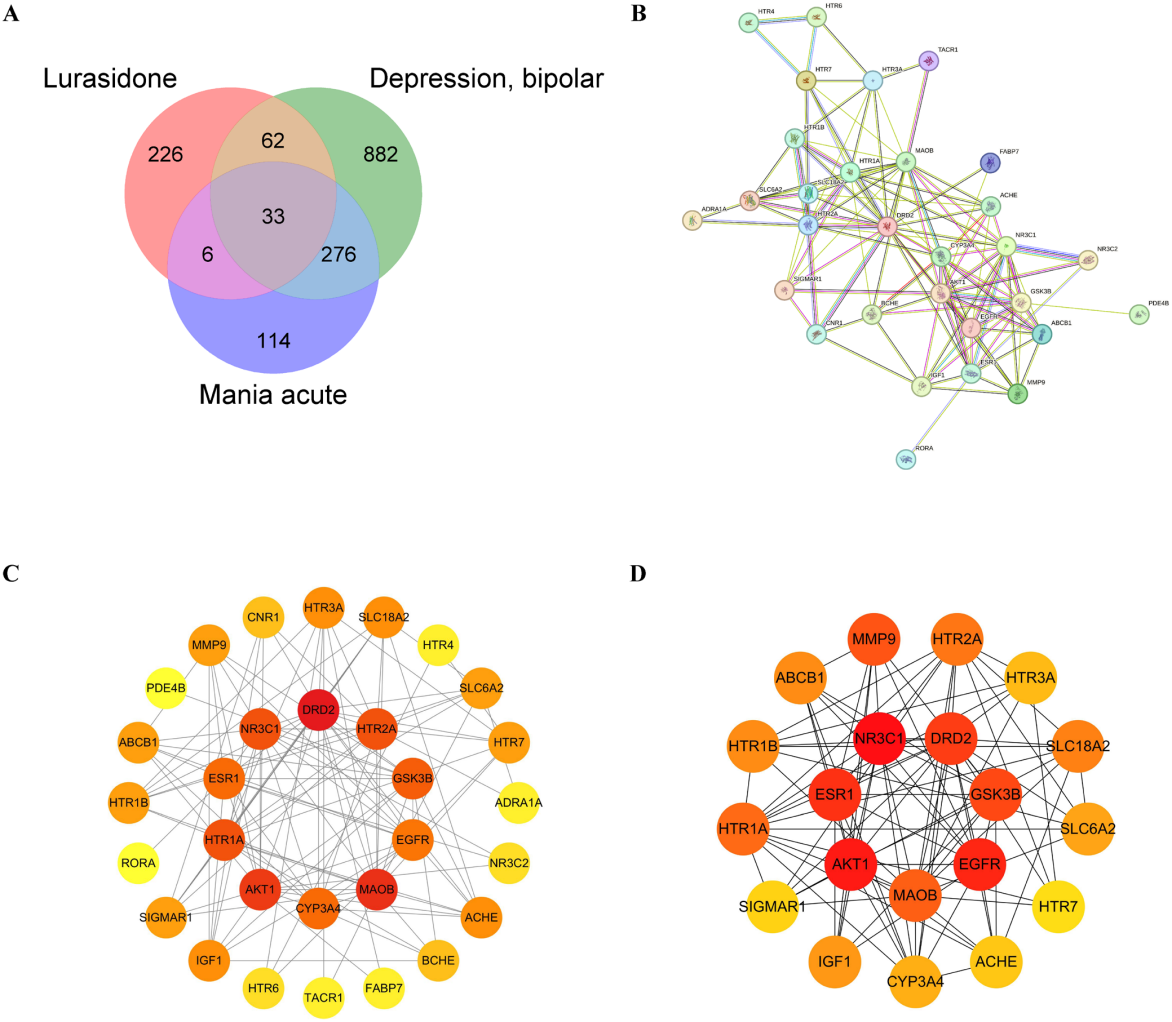
PPI network of targets in common between lurasidone, bipolar depression, and acute mania. (A) Venn diagram portraying the intersecting drug‐disease targets. (B) The original PPI network downloaded from the STRING database with 30 nodes and 116 edges. (C) The PPI network diagram obtained using Cytoscape software. Node colors change from yellow to red, indicating an increase in node degree. (D) The top 20 hub targets in the PPI network screened by the MCC method using the CytoHubba plugin. MCC, maximal clique centrality.

Screening of the GeneCards, PharmMapper, SwissTargetPrediction, and DrugBank databases yielded 8, 232, 112, and 6 lurasidone targets, respectively. After eliminating duplicates, a total of 327 potential drug‐related targets was obtained. Screening of the DisGeNET and GeneCards databases yielded 116 and 1216 bipolar depression targets and 2 and 428 acute mania targets, respectively. Removing duplicate genes resulted in a total of 1253 potential targets for bipolar depression and 429 potential targets for acute mania. As portrayed in the Venn diagram, 33 common targets at the intersection of lurasidone, bipolar depression, and acute mania were identified (Figure 2A).

### 3.2 PPI Network to Identify the Hub Targets

To construct the PPI network, the 33 predicted targets in common between lurasidone, bipolar depression, and acute mania were first uploaded into the STRING database (Figure 2B). The PPI data were then imported into Cytoscape 3.10.0 for visualization and analysis of the protein interactions, resulting in the construction of a novel PPI network. The novel PPI network of lurasidone‐induced acute mania in bipolar depression included 30 nodes and 116 edges (Figure 2C). Yellow to red node colors indicate increasing node degrees in the novel PPI network. The CytoHubba plug‐in was used to select the top 20 hub targets (Figure 2D).

### 3.3 GO Enrichment Analysis

**FIGURE 3:**
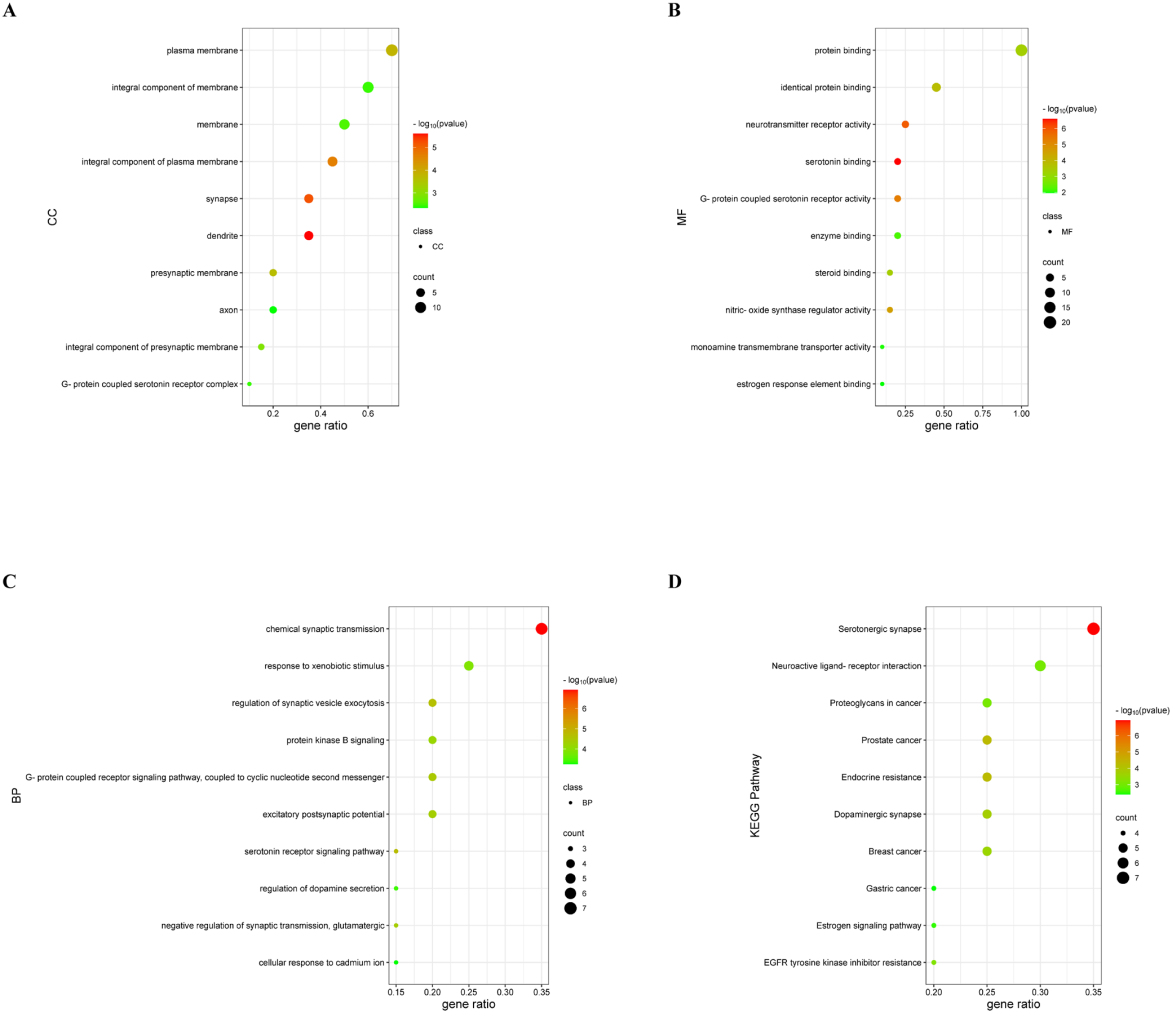
GO and KEGG enrichment analyses of the hub targets. (A) The top 10 cellular components (CCs). (B) The top 10 molecular functions (MFs). (C) The top 10 biological processes (BPs). (D) The top 10 KEGG signaling pathways. The color and size of each bubble indicate the *p* value and gene count, respectively. GO, Gene Ontology; KEGG, Kyoto Encyclopedia of Genes and Genomes.

The GO enrichment analysis of the top 20 hub targets of lurasidone‐induced acute mania in people with bipolar depression revealed 15 CCs, 23 MFs, and 88 BPs. The top 10 enriched CC, MF, and BP terms are presented as bubble charts (Figure 3A–C). The highly enriched CC terms included dendrite, synapse, integral component of plasma membrane, and presynaptic membrane. The highly enriched MF terms included serotonin binding, neurotransmitter receptor activity, and G protein–coupled serotonin receptor activity. The highly enriched BP terms included chemical synaptic transmission, serotonin receptor signaling pathway, and regulation of synaptic vesicle exocytosis.

### 3.4 KEGG Pathway Analysis and Drug‐Pathway‐Target‐Disease Network

To explore the potential mechanism behind lurasidone‐induced acute manic episodes in bipolar depression, the top 20 hub targets were imported into the Database for Annotation, Visualization and Integrated Discovery for KEGG pathway analysis. Based on the enrichment results of the hub targets, the top 10 significantly enriched pathways ranked by *p* value were selected (Table 2 and Figure 3D). The pathways with the top 3 highest gene counts were serotonergic synapse, neuroactive ligand‐receptor interaction, and dopaminergic synapse signaling pathways. The serotonergic synapse signaling pathway had the highest number of hub target counts, indicating that lurasidone may act via this pathway to induce acute manic episodes in people with bipolar depression.

**TABLE 2:** KEGG enrichment results of core targets in lurasidone‐induced acute manic episodes in people with bipolar depression.

ID	Term	Count	Gene	*p*
hsa04726	Serotonergic synapse	7	HTR7, MAOB, HTR1A, HTR1B, HTR3A, HTR2A, and SLC18A2	1.15E−07
hsa05215	Prostate cancer	5	GSK3B, AKT1, IGF1, MMP9, and EGFR	5.08E−05
hsa01522	Endocrine resistance	5	AKT1, IGF1, ESR1, MMP9, and EGFR	5.28E−05
hsa04728	Dopaminergic synapse	5	GSK3B, MAOB, AKT1, DRD2, and SLC18A2	1.69E−04
hsa05224	Breast cancer	5	GSK3B, AKT1, IGF1, ESR1, and EGFR	2.55E−04
hsa01521	EGFR tyrosine kinase inhibitor resistance	4	GSK3B, AKT1, IGF1, and EGFR	6.41E−04
hsa05205	Proteoglycans in cancer	5	AKT1, IGF1, ESR1, MMP9, and EGFR	9.00E−04
hsa04080	Neuroactive ligand–receptor interaction	6	HTR7, HTR1A, HTR1B, HTR2A, DRD2, and NR3C1	9.53E−04
hsa04915	Estrogen signaling pathway	4	AKT1, ESR1, MMP9, and EGFR	0.003133744
hsa05226	Gastric cancer	4	GSK3B, ABCB1, AKT1, and EGFR	0.00397189

Abbreviation: KEGG, Kyoto Encyclopedia of Genes and Genomes.

**FIGURE 4:**
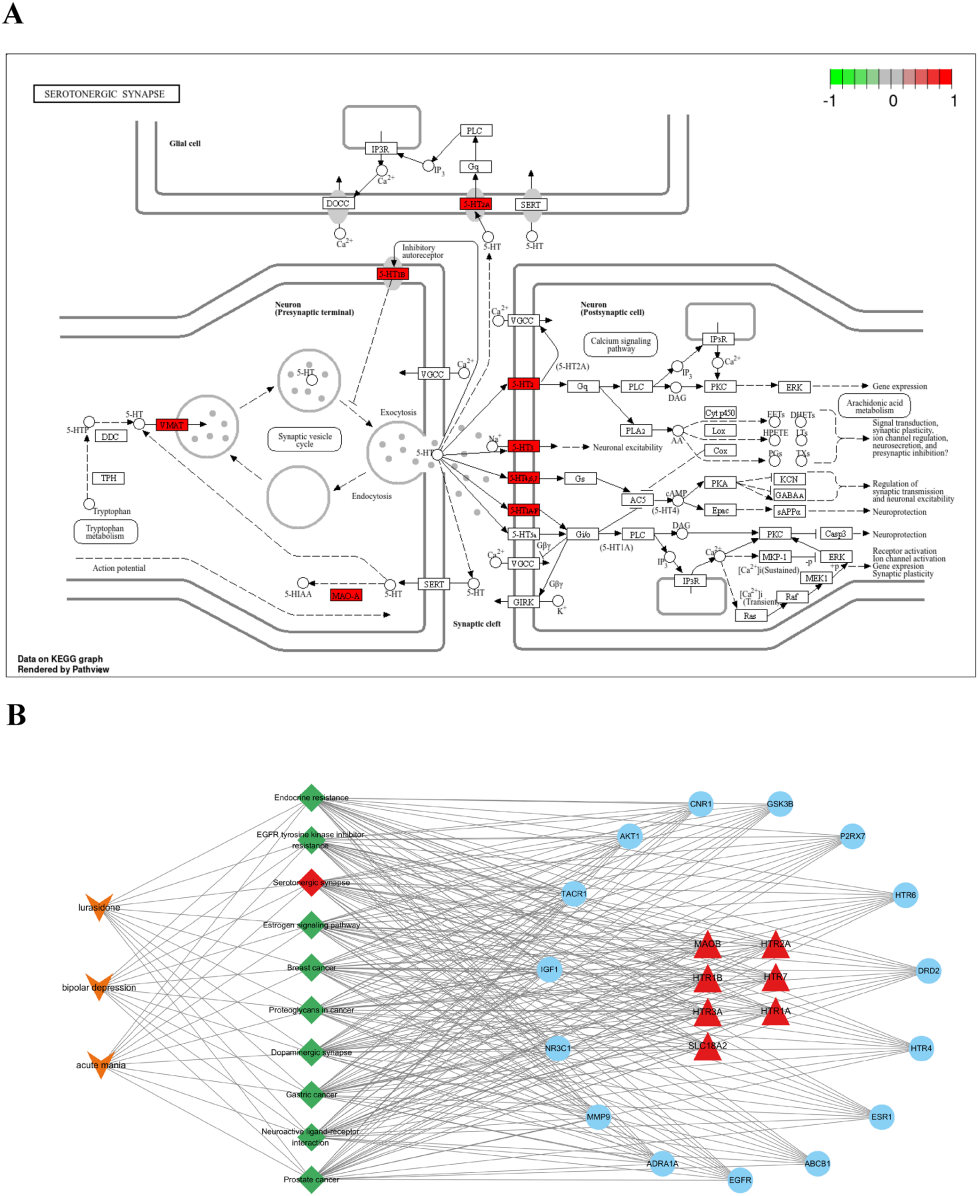
The distribution of the seven core targets in the serotonergic synapse signaling pathway and the drug‐pathway‐target‐disease network. (A) The distribution of the seven core targets in the serotonergic synapse signaling pathway. (B) The drug‐pathway‐target‐disease network diagram of lurasidone‐induced acute mania in people with bipolar depression.

The drug‐pathway‐target‐disease network was constructed using Cytoscape 3.10.0 (Figure 4B) and included 35 nodes (1 drug, 10 pathways, 22 targets, and 2 diseases) and 250 edges. The core targets for the potential effects of lurasidone‐induced acute mania in people with bipolar depression in the serotonergic synapse signaling pathway are presented in Figure 4A.

### 3.5 Molecular Docking Verification

**FIGURE 5:**
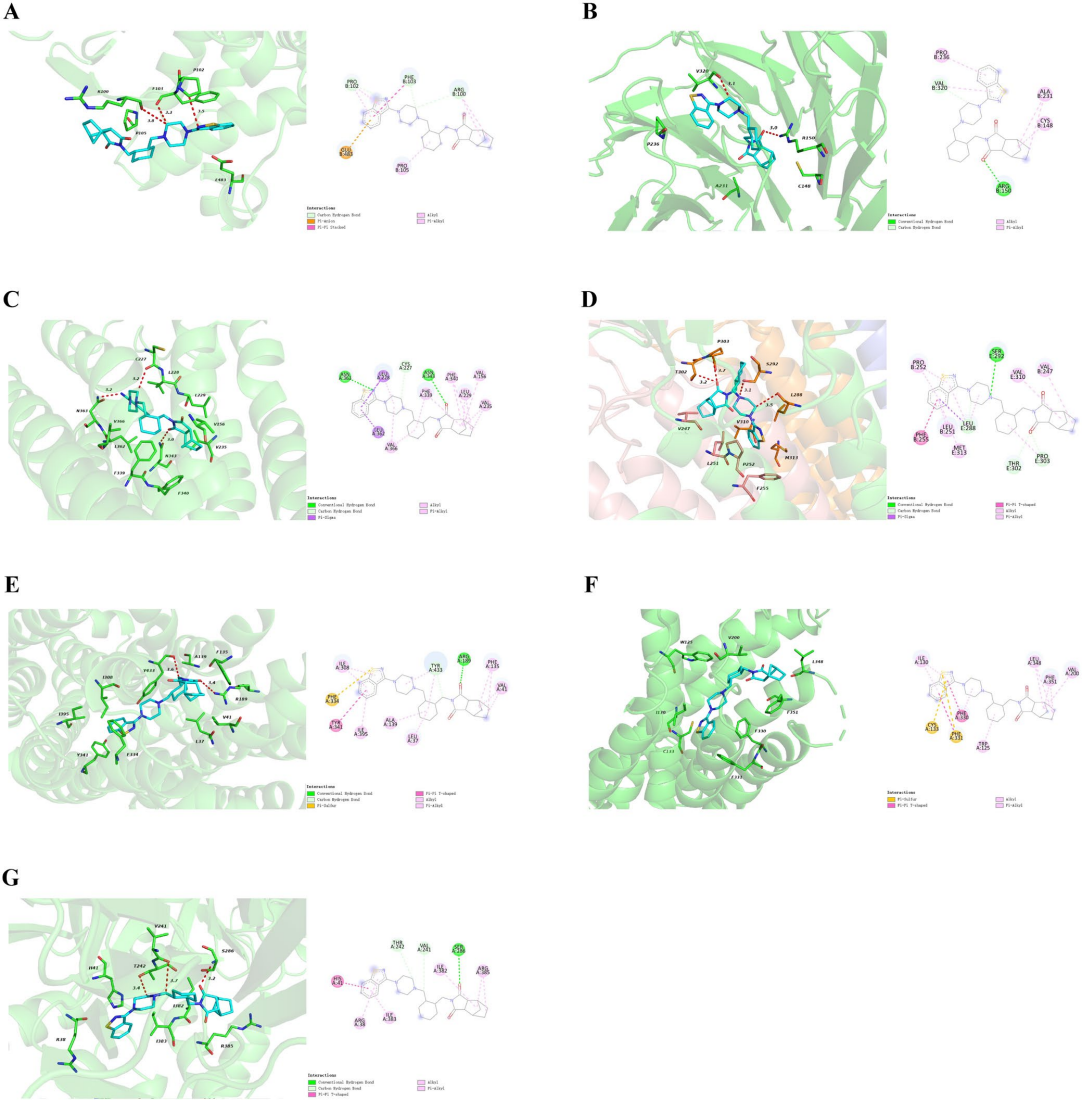
Docking patterns of lurasidone and core targets. (A) Lurasidone and MAOB. (B) Lurasidone and HTR1A. (C) Lurasidone and HTR2A. (D) Lurasidone and HTR3A. (E) Lurasidone and SLC18A2. (F) Lurasidone and HTR1B. (G) Lurasidone and HTR7. The interactions between lurasidone and the core proteins were visualized using Discovery Studio 2021 (2D) and PyMOL 2.4.1 (3D). The red dotted line in the 3D map represents a hydrogen bond.

The core targets of lurasidone‐induced acute mania in people with bipolar depression were mainly enriched in the serotonergic synapse signaling pathway (Figure 3D). Network pharmacological analysis identified seven core targets enriched in the serotonergic synapse signaling pathway: MAOB, HTR1A, HTR2A, HTR3A, SLC18A2, HTR1B, and HTR7. Details of the seven core targets are presented in Table 3. The interactions between lurasidone and the seven core target proteins were visualized using Discovery Studio 2021 (2D) and PyMOL 2.4.1 (3D) (Figure 5A–G). Docking scores ranged from −7.5 to −11.4 kcal/mol (Table 4), indicating good binding affinities between lurasidone and the core targets. Lurasidone had the highest binding affinities (−11.4 kcal/mol) for both HTR2A and HTR1B. The binding affinities between lurasidone and HTR2A were attributed to hydrogen bonding with ASN‐343, ASN‐363, and CYS‐227 residues and hydrophobic interactions with LEU‐228, LEU‐229, LEU‐362, VAL‐156, VAL‐235, VAL‐366, PHE‐339, and PHE‐340 residues (Figure 5C). The binding affinities between lurasidone and HTR1B were attributed to hydrophobic interactions with PHE‐330, PHE‐331, PHE‐351, VAL‐200, LEU‐348, TRP‐125, and ILE‐130 and other interactions with CYS‐133 and PHE‐331 (Figure 5F). Taken together, these data indicate that lurasidone could bind well with the seven selected core targets, and these interactions may play a key role in lurasidone‐induced treatment‐emergent mania in people with bipolar depression.

**TABLE 3:** Information on the seven core targets.

No.	UniProt ID	Gene symbol	PDB ID	Protein name	Degree
1	P27338	MAOB	1S3E	Monoamine Oxidase B	16
2	P08908	HTR1A	7E2Y	5‐Hydroxytryptamine Receptor 1A	13
3	P28223	HTR2A	7WC8	5‐Hydroxytryptamine Receptor 2A	13
4	P46098	HTR3A	8AXD	5‐Hydroxytryptamine Receptor 3A	8
5	Q05940	SLC18A2	8JSW	Solute Carrier Family 18 Member A2	8
6	P28222	HTR1B	4IAQ	5‐Hydroxytryptamine Receptor 1B	7
7	P34969	HTR7	7XTC	5‐Hydroxytryptamine Receptor 7	7

Abbreviation: PDB, Protein Data Bank.

**TABLE 4:** Molecular docking scores (kcal/mol).

Ligand	Receptor						
	MAOB	HTR1A	HTR2A	HTR3A	SLC18A2	HTR1B	HTR7
Lurasidone	−7.5	−10.4	−11.4	−10.4	−11.0	−11.4	−10.2

## 4 Discussion

It is well‐established that bipolar depression is a challenge to treat, as antidepressant use to this effect often induces manic episodes. While several antipsychotics have been approved for treating bipolar depression, treatment with these medications can still induce manic‐switch episodes, with several recent studies reporting acute manic episodes in people with bipolar depression treated with lurasidone. Despite this, the exact mechanism by which this occurs remains unclear. This study aimed to investigate potential targets and pathways involved in lurasidone‐induced acute manic episodes in people with bipolar depression. Information on the targets of lurasidone, bipolar depression, and acute mania and associated pathways was integrated with public data to establish a multilevel biological drug‐pathway‐target‐disease network. Based on this network pharmacology analysis, potential mechanisms underlying lurasidone‐induced acute manic episodes in people with bipolar depression were explored.

Lurasidone is a mood‐stabilizing atypical antipsychotic with a unique receptor‐binding profile. Like other second‐generation antipsychotics, lurasidone has full antagonistic properties at the D_2_ and 5‐HT_2A_ receptors. This is significant, as a favorable D_2_/5‐HT_2A_ balance may result in a lower propensity for extrapyramidal symptoms [44]. The initial antidepressant effects of lurasidone in people with bipolar depression are associated with its ability to reduce norepinephrine inhibition through its agonistic activity at 5‐HT_1A_ receptors and antagonistic activity at alpha‐1 norepinephrine and serotonin 5‐HT_2A_ receptors [45, 46]. The 5‐HT_7_ receptor is highly enriched in the hippocampus, limbic system, amygdala, and prefrontal cortex, and numerous studies have shown that it is involved in complex cognitive processes and mood regulation [21, 47, 48, 49].

A large lurasidone study found that 3.7% of patients receiving 20–60 mg of lurasidone experienced treatment‐emergent mania, which was higher than the 1.9% observed in both patients receiving 80–120 mg lurasidone and patients receiving placebo [50]. One possible reason for this inverse dose–response relationship is that lower doses of lurasidone may exert greater serotonergic effects, potentially due to receptor desensitization [51], conformational changes [52], or biased signaling [53]. Several studies have demonstrated that different concentrations of ligands can stabilize distinct receptor conformations, potentially shifting receptors between active and inactive states. For example, one previous study [54] showed that the aryloxypropanolamines CGP 12177 and LY 362884 can differentially stabilize distinct β1‐adrenergic receptor conformations in a concentration‐dependent manner. While β1‐adrenergic and 5‐HT receptors belong to different G protein‐coupled receptor subfamilies, a similar concentration‐dependent conformational stabilization may also occur with lurasidone. Lurasidone has a higher affinity for the 5‐HT_7_ receptor compared to other atypical antipsychotics. Thus, another possible hypothesis is that lurasidone's high 5‐HT7 receptor antagonism may not only contribute to its efficacy in bipolar depression, but could also play a role in its potential to induce mania, a hypothesis that warrants further investigation.

To better understand how treatment with lurasidone induces mania, computational and bioinformatics approaches were employed. Screening for targets common to lurasidone, bipolar depression, and acute mania yielded 33 targets potentially involved in lurasidone‐induced acute manic episodes in people with bipolar depression. A PPI network based on these results was constructed, and the top 20 hub targets were screened. Based on the PPI network and KEGG pathway analyses, MAOB, HTR1A, HTR2A, HTR3A, SLC18A2, HTR1B, and HTR7 were selected as core targets. While HTR1A, HTR2A, and HTR7 are known targets of lurasidone, MAOB, HTR3A, SLC18A2, and HTR1B were identified for the first time as targets of lurasidone potentially involved in its associated induction of acute manic episodes in people with bipolar depression.

A study conducted in pig brain cortex mitochondria demonstrated that four novel antipsychotics, brexpiprazole, cariprazine, loxapine, and lurasidone, all partially inhibited MAOA. Out of these four, only brexpiprazole and loxapine acted as partial inhibitors of MAOB, suggesting that MAOA inhibition may contribute to antidepressant effects while MAOB inhibition may promote neuroplasticity and neuroprotection [55]. The SLC18A2 gene encodes vesicular monoamine transporter (VMAT) 2, which is involved in storing and releasing tardive dyskinesia‐related monoamines, such as serotonin, norepinephrine, and dopamine [56]. The VMAT gene may play a role in genetic predisposition to both schizophrenia and bipolar disorder [57]. One study found that the overexpression of VMAT2 in mice resulted in increases in vesicle capacity, striatal dopamine content and release, and motor activity [58].

Several clinical and animal studies have indicated that 5‐HT_3_ receptor antagonists are effective in the treatment of depression, anxiety, irritable bowel syndrome, Alzheimer's disease, and antipsychotic‐associated tardive dyskinesia [59, 60, 61, 62]. A review found an apparent association between 5‐HT_3A_ polymorphisms and bipolar affective disorder in Caucasians that was also associated with the presence of decreased harm avoidance in women [63]. Chronic administration of the selective serotonin reuptake inhibitors (SSRIs) paroxetine, fluoxetine, and vortioxetine has been shown to enhance 5‐HT tone by diminishing the function of terminal 5‐HT_1B_ autoreceptors [64, 65]. Asenapine, an atypical antipsychotic, enhances 5‐HT neurotransmission by acting as a partial agonist of the 5‐HT_1A_ and 5‐HT_1B_ receptors [66]. It has also been reported to have antimanic properties and to be effective in vivo in antagonistic activity at 5‐HT1A/7 receptors [67]. Our network pharmacology‐based analysis suggests that lurasidone‐induced manic switching in bipolar depression may be associated with the modulation of HTR1A and HTR7, possibly through a mechanism similar to that of asenapine.

GO enrichment analysis revealed many biological processes associated with lurasidone‐induced acute manic episodes in people with bipolar depression, including chemical synaptic transmission, serotonin receptor signaling pathway, and regulation of synaptic vesicle exocytosis. The hub targets in this study were enriched in neurotransmitter‐related pathways, such as serotonergic synapse, neuroactive ligand‐receptor interaction, and dopaminergic synapse signaling pathways. The serotonergic synapse signaling pathway is the most likely pathway in the network to be associated with lurasidone‐induced acute mania in people with bipolar depression, and it may be associated with synaptic plasticity, regulation of synaptic transmission, and neuronal excitability, ion channel activation, and neuroprotection.

The induction of depression and hypomania/mania episodes in bipolar disorder is a complex process, with many physiological pathways involved. Our previous study revealed that the pharmacological mechanisms of quetiapine against bipolar depression and mania may be different. Whereas quetiapine affects bipolar depression via the MAPK and PI3K/AKT insulin signaling pathways, it affects bipolar mania through the neuroactive ligand‐receptor interaction signaling pathway [29]. It has been shown that elevated catecholamines can trigger manic episodes in patients with bipolar disorder through increased sensitivity of dopamine and norepinephrine receptors [68]. People who developed treatment‐emergent hypomania/mania after receiving SSRI treatment had an overrepresentation of the short allele polymorphism of the serotonin transporter [69]. While atypical antipsychotic‐induced mania is rare, it has been reported, with the antipsychotics lurasidone and cariprazine among those found to induce manic episodes in people with bipolar disorder [24, 70]. Both lurasidone and cariprazine are partial agonists to serotonin, and this, combined with the results of our computational biology analyses, indicates that the serotonergic synapse signaling pathway is potentially the pathway by which lurasidone treatment induces acute manic episodes in people with bipolar depression.

Molecular docking, a tool with extensive applications in computer‐aided drug design, is often used to explore the interaction sites between small molecules and macromolecules. To verify the accuracy of our network pharmacology results, we performed molecular docking analyses between lurasidone and potential target proteins. The binding mechanisms and affinities between lurasidone and the seven identified core target proteins within the serotonergic synapse signaling pathway (MAOB, HTR1A, HTR2A, HTR3A, SLC18A2, HTR1B, and HTR7) were characterized. Docking scores between lurasidone and the core target proteins ranged from −7.5 to −11.4 kcal/mol, indicating good binding affinities. HTR2A and HTR1B exhibited the highest binding affinities among the seven target proteins, suggesting that these proteins may be the ones largely responsible for lurasidone‐induced treatment‐emergent mania in people with bipolar depression.

This study, by combining network pharmacology and molecular docking, provides a novel approach for discerning the molecular mechanism underlying lurasidone‐induced acute manic episodes in people with bipolar depression. However, there are still several limitations that need to be addressed and overcome. First, the reliability and accuracy of predictions for target data from databases depend on the quality of the data; therefore, regular updates are required to maintain data precision. Second, previous case reports have demonstrated dosage is a key factor in lurasidone‐induced treatment‐emergent mania, and the current network pharmacology approach is limited to qualitative analysis of drug targets and lacks quantitative analysis of dose‐related effects. Finally, this study used an approach based on data mining and bioinformatics. Clinical trials and animal experiments will be necessary in order to verify these findings.

## 5 Conclusions

This study is the first to systematically explore the molecular mechanisms underlying lurasidone‐induced acute manic episodes in people with bipolar depression using network pharmacology and molecular docking approaches. Our bioinformatics and computational analyses demonstrated that lurasidone may regulate the serotonergic synapse signaling pathway by targeting proteins such as MAOB, HTR1A, HTR2A, HTR3A, SLC18A2, HTR1B, and HTR7 to induce treatment‐emergent mania in people with bipolar depression. These findings will raise awareness of the risk of lurasidone‐induced manic episodes among clinicians and emphasize the importance of careful monitoring of bipolar patients. By enhancing current understandings of lurasidone's potential adverse effects, this study contributes to the safer clinical use of this drug and helps inform decision‐making in the management of bipolar depression.

## Author Contributions

Conceptualization: Chuanjun Zhuo. Data curation: Chao Li and Hongjun Tian. Methodology: Chao Li, Lei Yang, Qiuyu Zhang, and Ying Zhang. Formal analysis: Chao Li and Chuanjun Zhuo. Investigation: Kaifang Yao, Lina Wang, Xiaoyan Ma, Ranli Li, and Feng Jia. Writing (original draft): Chao Li. Writing (review and editing): Chao Li and Chuanjun Zhuo. All authors have read and approved the final manuscript.

## Conflicts of Interest

The authors declare no conflicts of interest.

## Data Availability

The data that support the findings of this study are available from the corresponding author upon reasonable request.
